# The relationship between cannabis and anorexia nervosa: a scoping review

**DOI:** 10.1186/s40337-023-00887-9

**Published:** 2023-10-19

**Authors:** Chloe I. Rogers, Carly R. Pacanowski

**Affiliations:** Department of Health Behavior and Nutrition Sciences, University of Delaware, Newark, Delaware, USA

**Keywords:** Cannabis, Endocannabinoids, Cannabinoid receptors, Cannabis use disorder, Cannabis harms, Anorexia nervosa

## Abstract

**Background:**

Relapse rates in Anorexia Nervosa (AN) remain high, warranting exploration of further treatments. Cannabinoid agonists are of interest as they have shown successful outcomes in the treatment of associated conditions, such as post-traumatic stress disorder. This scoping review explores the endocannabinoid system (ECS), benefits/harms/null effects of cannabinoid treatment, and harms of cannabis use in AN.

**Methods:**

PubMed, PsycINFO, Cochrane, and Web of Science were searched for studies published between 2010 and August 2023, with human participants that explored the ECS, cannabinoid treatment, or cannabis use, and included 1 or more keywords for both cannabis and AN in the title and or abstract. Reports describing secondary anorexia, reports not available in English, grey literature, reports combining data from AN with other conditions, and reports only reporting the prevalence of cannabis abuse/dependence were excluded. Data were extracted from 17 reports (n = 15 studies). For the ECS, outcomes included genetics such as allele expression related to the ECS, cannabinoid receptor availability, and circulating levels of endocannabinoids. For benefits/harms/null effects of cannabinoid treatment, outcomes included changes in weight, eating disorder (ED) symptoms, physical activity (PA), and hormones. For harms of cannabis use, outcomes included genetics related to cannabis use disorder and associations between cannabis use and ED symptoms.

**Results:**

Eight studies (n = 8 reports) found abnormalities in the ECS in AN including expression of related alleles, genotypes, and haplotypes, availability of cannabinoid receptors, and levels of endocannabinoids. Three studies (n = 5 reports) found benefits/harms/null effects of cannabinoid treatment. Benefits included weight gain, improved ED symptoms and reduced PA, while null effects included no changes in weight or ED symptoms, and harms included increased PA and lowered adipose hormones. Four studies (n = 4 reports) expanded upon harms of cannabis use, including genetic predispositions to cannabis use disorder, and compensatory behaviors related to cannabis use.

**Conclusion:**

Limited evidence suggests that abnormalities in the ECS in AN may render cannabis a potential treatment for weight restoration and associated symptoms. Future research may wish to investigate individualized dosing approaches to maximize beneficial effects while minimizing harms.

Level II Evidence: Scoping Review.

**Supplementary Information:**

The online version contains supplementary material available at 10.1186/s40337-023-00887-9.

## Abstract (es)

Anorexia Nervosa (AN) affects people from various backgrounds causing notable physical and mental impairments. A recovery process that is successful for everyone who has the condition does not exist. Due to high relapse rates in AN, exploring further treatments is imperative. Cannabis has shown promise in treatments of other psychiatric disorders, some of which also occur in those with AN; thus, an overview of the available research is warranted. This scoping review presents results from studies about the relationship between cannabis and AN. Results suggest that individuals with AN have abnormalities in a biological system that interacts with cannabis, proposing the potential usefulness of cannabis for recovery. Although some studies reported benefits of cannabis for AN, including weight gain, improved eating disorder (ED) symptoms, and reduced physical activity (PA), other studies suggested no changes in weight or ED symptoms, increased PA, and worsened appetite hormone levels. Lastly, some studies suggested that individuals at higher risk of developing AN may be at greater risk for developing cannabis use disorder, and that cannabis use may be associated with ED symptoms. Future studies should examine individualized dosing of cannabis in AN to maximize benefits and minimize harms.

## Background

Anorexia nervosa (AN) is a life-threatening eating disorder (ED) with high relapse rates [1]. Given the uncertain ability of current psychiatric treatments to fully address AN symptoms, exploring alternative treatments is warranted. [2]. Current treatment options for AN include psychotherapy (e.g., cognitive-behavioral therapy) and pharmacotherapy (e.g., antidepressants and antipsychotics) [3]. Although these treatment options have brought upon beneficial outcomes such as weight gain and reduced urge for compensatory behaviors, they do not appear to completely address the disturbances in appetite during AN recovery. For example, appetite signals may be distorted in AN during and after treatment [4, 5].

Cannabinoids, the chemical components of the cannabis plant, are of interest for AN as they not only have the potential to increase appetite, but also reduce anxiety [6, 7]. Cannabinoids have also been associated with improved symptoms of co-occurring conditions of AN, such as post-traumatic stress disorder [7]. Thus, further exploration of cannabinoids is warranted for AN treatment.

Cannabinoids bind with receptors located throughout the central and peripheral nervous systems, where they carry out psychological and physiological functions such as reduced anxiety, appetite stimulation, and pain relief [6]. The 2 most well-understood cannabinoids are ∆9-tetrahydrocannabinol (∆9-THC) and cannabidiol (CBD) [8]. ∆9-THC is responsible for the psychoactive effects of cannabis and has effects on mood, appetite, pain, and memory [7]. CBD can reduce anxiety, inflammation, and nausea, and can protect neuronal cells. ∆9-THC and CBD bind with cannabinoid receptors located in the endocannabinoid system (ECS) to carry out these functions [9]. The ECS is a lipid communication network that plays roles in several psychological and physiological processes. Cannabinoid receptors (CB1 and CB2) are G-protein coupled receptors within the ECS that allow for the binding of ∆9-THC, CBD, and naturally occurring circulating endocannabinoids. The 2 most well-understood endocannabinoids are N-arachidonoylethanolamine (AEA), and 2-arachidonoylglycerol (2-AG). Both AEA and 2-AG are derived from polyunsaturated fatty acids and play major roles in the brain. Studies have found that EDs are associated with abnormalities in the ECS and therefore, have been suggested to serve as a potential biomarker [10].

The CB receptors are located throughout central and peripheral points such as the hypothalamus, ultimately playing roles in food intake [11]. Consequently, the ECS influences one’s appetite and pleasure associated with eating. As mentioned previously, there is evidence suggesting a dysregulation of appetite signaling in AN, leading to difficulties meeting caloric needs during recovery [4, 5]. Thus, cannabinoid treatment may assist in AN recovery as cannabinoids interact with the ECS, and may modify abnormalities, ultimately improving appetite regulation and caloric intake [9, 10].

When considering cannabinoid treatment for AN, it is important to consider potential adverse outcomes. Cannabis is a psychoactive substance and unhealthy use is possible. A recent meta-analysis found that substance use disorders are prevalent amongst individuals with AN which may pose risks of dependence when considering cannabinoid treatment [12]. Indeed, this meta-analysis found a 6% prevalence of cannabis abuse/dependence in those with AN, with most individuals falling under the binge/purge AN subtype. Adverse effects of cannabis use, which may exist beyond abuse and dependence, should be considered. For example, long-term cannabis use has been associated with a cyclic vomiting condition known as cannabinoid hyperemesis syndrome (CHS) [13]. Exploring adverse effects of cannabis such as CHS is warranted as it may inform safer practices when initiating cannabinoid treatment for those with AN.

An existing review regarding cannabinoid treatment in AN was limited to randomized controlled trials (RCTs) and did not include information about the ECS and harms associated with cannabis use [14]. Reviewing all levels of evidence exploring cannabinoid treatment in AN is warranted as findings from studies other than RCTs may prompt development of hypotheses for future research. Furthermore, reviewing studies exploring the ECS and harms associated with cannabis use may suggest biological justification for treatment and inform safer practices regarding cannabinoid interventions for AN. For these reasons, the purpose of this scoping review is to provide an overview of literature addressing the following questions: 1.) What are the differences between the ECS in participants with AN and healthy controls (HC)? 2.) What are the benefits/harms/null effects of cannabinoid treatment for AN? and 3.) What are the harms associated with cannabis use in individuals with AN?

## Methods

### Guidelines

The Joanna Briggs Institute guidelines were used to complete this scoping review [15]. To ensure the adherence to these guidelines, the Preferred Reporting Items for Systematic Reviews and Meta-Analyses extension for Scoping Reviews (PRISMA-ScR) checklist [16] was used and can be found in the Additional file 1. Screening of records and full-text reports, data extraction, and data analysis were completed by CIR.

### Literature retrieval

PubMed, PsycINFO, Cochrane Library, and Web of Science databases were searched for peer-reviewed literature describing relationships between cannabis and AN. Each search was conducted until August 1^st^, 2023 and included reports published between the years 2010–2023. Full searches used for each database can be found in the Additional file 2. Although the full searches for each database differed slightly due to the availability of Medical Subject Headings (MeSH terms) in some databases and not others, all searches contained the same keywords. Duplicates were identified and removed using Zotero software. Titles and abstracts were screened, and either accepted or eliminated according to inclusion/exclusion criteria.

**Fig. 1:**
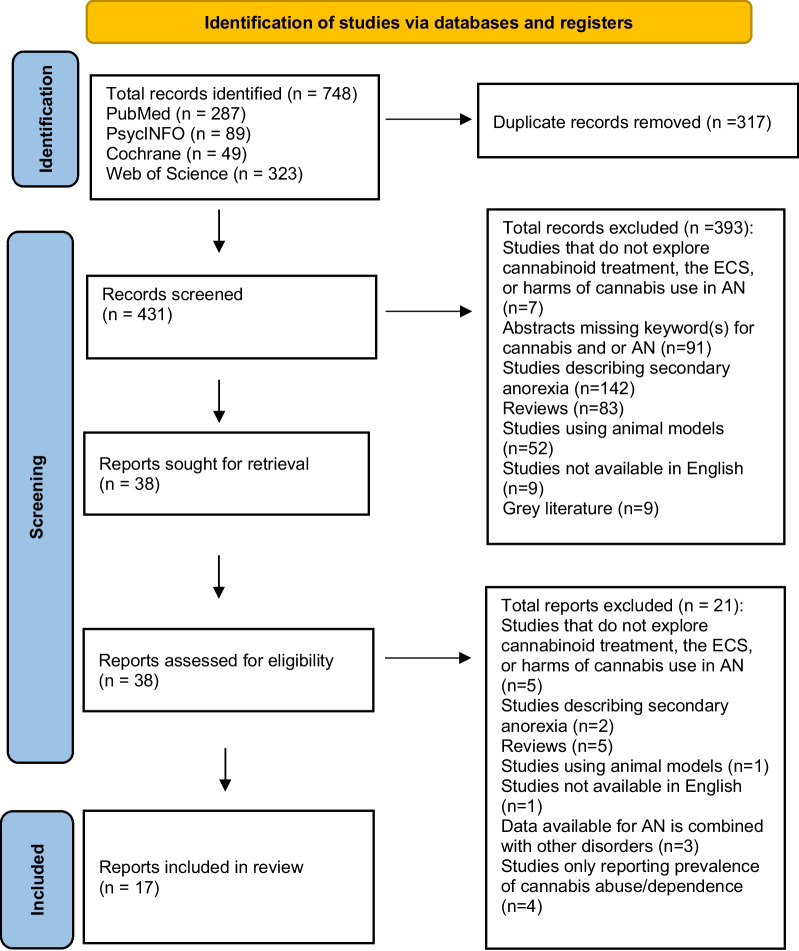
PRISMA-ScR diagram

The Population, concept, and context (PCC) statements were used to guide the inclusion of reports [15]. For this scoping review, the population consisted of humans with AN, the concept consisted of the ECS, benefits/harms/null effects of cannabinoid treatment, and harms associated with cannabis use, with a context open to any setting. To be considered eligible, reports had to include human participants, explore the ECS in AN, cannabinoid treatment in AN, or harms associated with cannabis use in AN, and include 1 or more keywords for both cannabis and AN in the title and or abstract. A complete list of keywords can be found in Table 1. Studies describing secondary anorexia, reviews, studies including animal models, studies written in languages other than English, grey literature, studies combining data from individuals with AN with data from individuals with other psychiatric disorders (including EDs other than atypical AN such that data specific to AN could not be extracted) and studies reporting the prevalence of cannabis abuse/dependence without presenting additional harms were excluded. For further details regarding the included/excluded reports, refer to the PRISMA-ScR diagram in Fig. 1.

**Table 1:** Keywords

Keywords for Cannabis	Keywords for AN
• Cannabis • Dronabinol • Cannabinol • Marinol • ∆9-Tetrahydrocannabinol • Cannabidiol • THC • CBD • Cannabinoids • Marijuana • Endocannabinoids • Endocannabinoid system • ECS • Cannabinoid receptors • Cannabinoid receptor 1 • Cannabinoid receptor 2 • CB1 • CB2 • Arachidonoylglycerol • Anandamide • Oleoylethanolamide • Palmitoylethanolamide • Cannabis abuse • Cannabis use disorder • CUD • Cannabis dependence • Marijuana abuse • Marijuana dependence • Cannabis harms • Cannabis adverse effects • Cannabis negative effects	• Primary anorexia • Anorexia nervosa • Anorexia • Atypical anorexia nervosa • Atypical anorexia

### Data extraction

Data relevant to the research questions were extracted from each report. Tables 2 and 3 include the following information from each report: study design, participant characteristics, methods (indicating the use of controls if applicable) and results relevant to the research questions.

**Table 2:** Endocannabinoid system

Authors (year)	Study design	Participants	Methods, Interventions, and observations	Outcomes of Interest
Ishiguro et al., 2011 [17]	Case–control	235 AN, 1244 HC (all female)	Observed single-nucleotide polymorphisms related to circulating endocannabinoids from blood samples	• Val195 allele of GPR55 was more frequent in AN compared to HC (odds ratio = 1.30, 95% Cl: 1.02–1.66, p < 0.04)
Ando et al., 2014 [18]	Case–control	376 RAN, 387 BPAN, and 605 HC (all female)	Extracted genomic deoxyribonucleic acid related to circulating endocannabinoids from blood samples	• 385A allele was less frequent in AN compared to HC (odds ratio = 0.799, 95% CI 0.653–0.976, p = 0.028) • 385A allele was less frequent in RAN compared to BPAN (odds ratio = 0.717, 95% CI: 0.557–0.922, p = 0.0094)
González et al., 2021 [19]	Case–control	221 AN, 396 HC (all female)	Screened single-nucleotide polymorphisms in CB1 & 2 from blood samples. Measured Symptom Checklist 90 Revised, Eating Disorders Inventory Test-2, and qualitative questionnaires	• genotype rs806369-TT and haplotype​ rs806368/rs1049353/rs806369 of CB1 were associated with lower weight in AN (40.55 ± 1.61 kg, vs. 45.47 ± 0.49 kg, mean difference = -4.92 kg, 95% CI: –7.74, – 1.46, p = 0.004) and BMI (16.09 ± 0.44 kg/m^2^, vs. 17.47 ± 0.15 kg/m^2^, mean difference = -1.38 kg/m^2^, 95% CI: -2.23, -0.33, p = 0.008) • rs806374 of CB1 were associated with ED behaviors in AN (98.89 ± 4.90 vs. 83.93 ± 4.18, mean difference = – 14.96, 95% CI: – 26.820, – 1.710, p = 0.027) • rs3003335 and rs6658703 of CB2 were associated with ED behaviors in AN (87.13 ± 3.66 vs. 104.69 ± 6.61, mean difference = 17.56, 95% CI: 2.153, 32.220, p = 0.026)
Gérard et al., 2011 [20]	Cross-sectional	14 AN, 19 HC (all female)	Performed positron emission tomography imaging to assess CB1 availability. Measured Eating Disorders Inventory Test and Eating Disorder Evaluation Scale	• CB1 availability was significantly higher in AN compared to HC (+ 24.5%, p = 0.0003; see Gérard et al. Figure 2B for supporting statistical values) • Positive association between CB1 availability and drive for thinness in AN (correlation coefficient = 0.86, p = 0.001)
Tam et al., 2021 [21]	Combined cross-sectional longitudinal	67 in UWAN state, 27 in WRAN state, 84 HC (all female)	Measured levels of endocannabinoids through hair samples in all participants during the cross-sectional period (T1). Levels of endocannabinoids were measured again in the longitudinal period (T2) in participants in UWAN who experienced ≥ 12% increase in BMI	AEA: • Elevated in UWAN (p = 0.021) and WRAN (p = 0.006) compared to HC in T1 • Decreased in UWAN during T2 compared to T1 (p = 0.022) 2-AG: • NS differences in T1 or T2 between groups OEA: • Elevated in UWAN compared to HC in T1 (p = 0.003) • Decreased in UWAN during T2 compared to T1 (p = 0.018) PEA; • Elevated in UWAN compared to HC in T1 (p = 0.018) • Decreased in UWAN during T2 compared with T1 (p = . 028) SEA: • Elevated in UWAN compared to WRAN in T1 (p = 0.018) • Elevated in UWAN compared to HC in T1 (p < 0.001) • Decreased in UWAN during T2 compared with T1 (p = 0.018) *See Tam et al. Figure 2 for supporting statistical values
Monteleone et al., 2015 [22]	Pre-meal/Post-meal study	7 in UWAN state [1 women, 6 man], 7 in WRAN state [2 women, 5 men], 7 HC [2 women, 5 men]	Measured levels of endocannabinoids through plasma after a 13-h fasting period, and 15 min and 120 min after eating for both hedonic and non-hedonic foods	AEA: • Progressively decreased after eating both foods (F [3, 18] = 17.99, p < ​​0.00001) consistent in all groups 2-AG: • NS differences in UWAN • Progressively increased after eating both hedonic and non-hedonic foods in WRAN (See Monteleone et al. Figure 2 for supporting statistical values) • Progressively decreased after eating hedonic foods compared to non-hedonic foods in HC (See Monteleone et al. Figure 2 for supporting statistical values) OEA: • Lower after eating hedonic foods compared to non-hedonic foods (F [1, 18] = 6.30, p = 0.02) consistent in all groups PEA: • Progressively decreased after eating hedonic and non-hedonic foods (see Monteleone et al. Figure 5 for supporting statistical values) consistent in all groups
Piccolo et al., 2019 [23]	Pre-meal/Post-meal study	15 in UWAN state, 10 in WRAN state, 9 HC *Gender/sex not reported	Measured levels of endocannabinoids through plasma after an 8-h fasting period, and 120 and 240 min after eating	AEA: • Lower in AN compared to HC at all time points (5.83 ± 0.44 pmol/ml vs. 2.85 ± 0.34 pmol/ml, p < 0.001) • Elevated in UWAN & WRAN during fasting (4.69 ± 0.73 pmol/ml vs. 2.85 ± 0.34 pmol/ml and 4.64 ± 0.51 pmol/ml vs. 2.81 ± 0.22 pmol/ml, p < 0.001) • Elevated in UWAN & WRAN after 240 min (1.70 ± 0.26 pmol/ml vs. 2.85 ± 0.34 pmol/ml and 1.48 ± 0.30 pmol/ml vs. 2.81 ± 0.22 pmol/ml, p < 0.05) 2-AG: • NS differences between participants or time points
Baenas et al., 2023 [24]	Cross-sectional	27 AN, 29 HC (all women)	Measured levels of endocannabinoids through plasma after a 12-h fasting period	AEA: • NS differences in AN compared to HC • Lower levels were associated with higher BMI and higher emotional dysregulation in AN 2-AG: • NS differences in AN compared to HC • Higher levels were associated with worsened psychopathological states in AN

*CI = Confidence intervals; p < 0.005 = Significant; NS = Not Significant; RAN = Restrictive type AN; BPAN = Binge/purge type AN; UWAN = Underweight AN; WRAN = Weight restored AN; T1 = Cross-sectional period; T2 = Longitudinal period; BMI = Body mass index

**Table 3:** Benefits/harms/null effects of cannabinoid treatment & harms of cannabis use

Author/year	Study design	Participants	Methods, interventions, & observations	Outcomes of interest
Andries et al., 2014 (a) [27]	Crossover RCT (Parent Trial)	24 women with AN	Participants were divided into 2 groups and given either 5 mg of dronabinol per day for 4 weeks or a placebo. After a 4-week break period, participants received the opposite intervention for 4 weeks. Measured weight weekly and Eating Disorder Inventory scores during the 1^st^ and 4^th^ week of each intervention period	Benefits: • Weight gain of 1.00 kg (95% CI: 0.40–1.62) during dronabinol vs. 0.34 kg (95% CI: 29.14-0.82) during placebo (p = 0.03 above placebo) Harms/Null Effects: • NS differences in eating disorder inventory scores
Andries et al., 2014 (b) [28]	Report from Andries et al., 2014 (a) Parent Trial	24 women with AN	Reported intensity and duration of PA measured through accelerometers worn by participants daily	Harms/Null Effects: • Increased moderate to hard PA intensity during dronabinol compared to placebo in inpatients (3,958.3 ± 789.2 accelerometer counts per minute vs. 3,732.6 ± 936.1 accelerometer counts per minute respectively, p = 0.04) • NS differences in PA intensity in outpatients • NS differences in PA duration in inpatients • Increased moderate to hard PA duration during dronabinol compared to placebo in outpatients (0.9 ± 0.6 h/day vs. 0.8 ± 0.6 h/day respectively, p = 0.02)
Andries et al., 2015 [29]	Report from Andries et al., 2014 (a) Parent Trial	24 women with AN	Reported bioactive IGF, IGF-1, IGFBP-2 and -3, leptin, and adiponectin measured through overnight fasted serum and cortisol measured through 24-h urine samples on the last day of each intervention period	Harms/Null Effects: • NS changes in bioactive IGF, IGF-1, or IGFBP -2/-3 • NS changes in leptin after controlling for age, weight, and cortisol • Decreased adiponectin (naturally transformed regression coefficient: -0.07, 95% CI: -0.11 to 0.04, p < 0.01) in a model including leptin during dronabinol • Decreased cortisol during dronabinol (naturally transformed regression coefficient: -0.19, 95% CI: -0.37 to 0.05, p = 0.04)
Avraham et al., 2017 [30]	Non-randomized study	10 females with AN	All participants received 1 mg THC for 1 week, then 2 mg THC for 3 weeks. Measured BMI, Eating Disorder Inventory, Eating Attitude Test, Beck Depression Inventory, Body Shape Questionnaire, and Spielberger State-Trait Anxiety Inventory at baseline and at the end of the intervention period	Benefits: • Decreased asceticism (10.00 ± 2.46 vs. 7.06 ± 1.61, p = 0.049) • Decreased depression (3.12 vs. 2.50, p < 0.049) • Increased body care (19.22 ± 1.87 vs. 20.22 ± 1.79, p = 0.02) Harms/Null Effects: • NS changes in BMI • NS changes in anxiety
Graap et al., 2017 [31]	Case report	1 male with AN	7.5–15 mg dronabinol (increased dose weekly) during a 6-week period. Measured BMI weekly, steps through a pedometer daily, PA urge through a subjective 0–6 scale daily, Eating Disorder Examination questionnaire at baseline and at the end of the intervention, and leptin and cortisol through dexamethasone-suppression-tests at baseline and at week 5 of the intervention	Benefits: • Weight gain after dronabinol (BMI 19.5 kg/m^2^ pretreatment vs 21.0 kg/m^2^ posttreatment) • Decreased ED symptoms after dronabinol (sum score 5.2 pretreatment vs. 2.0 posttreatment) • Decreased PA levels after dronabinol (32,510 steps per day pretreatment vs. 17,493 steps per day posttreatment) • Decreased PA urge after dronabinol (average urge to move rating of 5 pretreatment vs. 3.5 posttreatment) Harms/Null Effects: • No changes in leptin • Dcreased cortisol levels during dronabinol (1.7 mg/dL pretreatment vs. < 1.0 mg/dL posttreatment)
Hjorthøj et al., 2019 [35]	Retrospective cohort study	*Number of participants with AN could not be determined	Investigated polygenic risk scores for AN and CUD from participants followed from birth (between years 1981–2001) until 2017	Harms: • Highest polygenic risk scores for AN were associated with CUD (Hazard Ratio = 1.41, 95% CI 1.27–1.56)
Ihm et al., 2023 [36]	Retrospective cohort study	16,992 individuals with AN, 55,525 HC *Gender/sex could not be determined	Investigated polygenic risk scores for AN and CUD from participants recruited between 2006–2010	Null effects: • NS interconnections between the polygenic risk scores for AN and polygenic risk scores for CUD
Brewerton et al., 2016 [37]	Case report	1 Woman with BPAN	Patient presented to a treatment center with AN relapse symptoms. A urinary drug test, physical exam, mental status exam, and laboratory analysis was taken upon admission	Harms: • Patient reported using cannabis daily for at least 1 year and chronically for at least 7 years • Patient reported vomiting, which was originally mistaken as a relapse • Patient reported relief in vomiting symptoms after hot showers, which lead to the diagnosis of CHS
Karayilan et al., 2013 [38]	Case report	1 Woman with BPAN	Patient presented to a treatment center with weight loss, restrictive eating, and nervosity. Physical examination and review of life and medical history were taken upon admission	Harms: • Patient reported using cannabis daily for about 3 years • Decrease in BMI over the course of 3 years of daily cannabis use (22.08 kg/m^2^ at the start of cannabis use vs 15.6 kg/m^2^ after 3 years) • Patient reported engagement in postprandial compensatory behaviors after using cannabis

*CI = Confidence intervals; p < 0.005 = Significant; NS = Not significant; IGF = Insulin-like growth factor; IGFBP = Insulin-like growth factor binding protein; BMI = Body mass index; BPAN = Binge/purge type AN

### Data analysis

Included reports were first grouped by topic (i.e., the ECS, benefits/harms/null effects of cannabinoid treatment, or harms of cannabinoid use). Data from each report were further grouped by outcomes to highlight similarities and discrepancies between results.

## Results

### Endocannabinoid system

Eight reports from 8 studies investigated the ECS in individuals with AN [17–24]. Study designs ranged from case control, combined cross-sectional longitudinal, pre-meal/post-meal, and cross-sectional, with sample sizes ranging from 14 to 763 participants with AN and 7 to 1244 healthy controls (HC). Most participants were female or identified as women, except for 5 participants who identified as men. One study did not report participants’ gender/sex [23]. Outcomes of interest included genetic factors related to the ECS (alleles, genotypes, and haplotypes) which code for CB receptors, and circulating endocannabinoids. Further details about these reports can be found in Table 2.

Since genetic factors such as alleles and genotypes code for protein receptors in the ECS, differences in these genetic factors may lead to discrepancies in the number of available protein receptors. Three case–control studies explored alleles, genotypes, or haplotypes related to the ECS. One study found that the Val195 allele of G-protein-coupled receptor-55, which has an affinity for endocannabinoids, was significantly more frequent in participants with AN compared to HC [17]. Another study found that the 385A allele, which codes for an enzyme that degrades the endocannabinoid, arachidonoylethanolamine (AEA) was significantly less frequent in AN compared to HC [18]. The remaining study found that in participants with AN, the genotype rs806369-TT and haplotype​ rs806368/rs1049353/rs806369 of CB1 were associated with significantly lower weight and body mass index (BMI) compared to HC [19]. Additionally, the genotypes of rs806374 for CB1 and rs3003335 and rs6658703 for CB2 were associated with significantly higher occurrences of ED behaviors compared to participants with AN not carrying said genotypes.

It has been suggested that circulating endocannabinoids play roles in food intake, energy expenditure, and possibly engagement in ED behaviors [11, 21–23]. Both availability of receptors (the percentage of receptors that are unbound) to which circulating endocannabinoids bind and levels of circulating endocannabinoids are important to consider. One cross-sectional study found that CB1 availability was significantly increased in AN and positively associated with drive for thinness [20].

Understanding circulating endocannabinoid levels in those with AN are important to determine whether exogenous cannabinoid treatment may be able to ameliorate symptoms of the disorder. Circulating endocannabinoid levels were measured in participants with AN through plasma or hair samples in 1 combined cross-sectional longitudinal study, 2 pre-meal/post-meal studies, and 1 cross-sectional study [21–24]. Endocannabinoids measured included AEA, 2-arachidonoylglycerol (2-AG), Oleoylethanolamide (OEA), palmitoylethanolamide (PEA), and Stearoylethanolamide (SEA) which are naturally occurring and circulate within cells, adipose tissue, muscles, and the brain [25].

Because endocannabinoids are related to food intake, levels could change in relationship to consumption and whether the food is liked (hedonic) or not liked (non-hedonic) [26]. Both pre-meal/post-meal studies accounted for time since meals, 1 of which also accounted for differences in hedonic/non-hedonic eating [22, 23]. Three out of the 4 studies categorized participants with AN as underweight (UWAN) or weight restored (WRAN) [21–23].

All 4 studies measured AEA, 2 of which found significant differences between AN and HC. One study found significantly elevated AEA in UWAN and WRAN compared to HC [21] while the other found significantly lower AEA in UWAN and WRAN compared to HC [23]. Another study found consistently lower AEA after eating both hedonic and nonhedonic foods compared to fasting periods in both AN and HC [22]. Lower AEA in AN was associated with higher BMI and higher emotional dysregulation in a separate study [24].

All 4 studies measured 2-AG, 1 of which found significant differences between WRAN and HC [22]. In WRAN, 2-AG was significantly elevated after eating both hedonic and non-hedonic foods compared to fasting periods. HC showed significantly lower 2-AG after eating hedonic foods compared to non-hedonic foods. In a separate study, higher 2-AG was associated with poorer psychological states in those with AN [24].

Only 2 studies measured OEA, 1 of which found significantly elevated OEA in UWAN compared to HC [21]. When these participants in UWAN became weight restored, they showed significantly lower OEA compared to their baseline, indicating the possibility that OEA levels may begin to present similar to HC upon weight restoration. The other study found significantly lower OEA after eating hedonic foods compared to non-hedonic foods, which was consistent for those with AN and HC [22].

PEA was measured in 2 studies, 1 of which found significantly elevated PEA in UWAN compared to HC [21]. When these participants in UWAN became weight restored, they showed significantly lower PEA compared to results from when they were underweight, indicating the possibility that levels of PEA may begin to present similar to HC with weight restoration. The other study found significantly lower PEA after eating both hedonic and non-hedonic foods compared to fasting periods, which was consistent in AN and HC [22].

Only 1 study measured SEA and found significantly elevated SEA in UWAN compared to WRAN and HC [21]. Further, UWAN showed significantly lower SEA upon weight restoration compared to their baseline, indicating the possibility that SEA may begin to present similar to HC upon weight restoration.

### Benefits/harms/null effects of cannabinoid treatment

Five reports from 3 studies including 1 crossover randomized controlled trial (RCT), 1 non-randomized study, and 1 case report explored cannabinoid treatment [27–31]. Cannabinoids were administered through capsules of THC or dronabinol, a synthetic form of THC [32]. Treatment dosages used in these reports ranged from 2 to 15 mg and length of intervention was between 4 and 6 weeks. Sample sizes ranged from 1 to 24 participants who were female or identified as women, except for 1 male from a case report. Outcomes measured included weight, ED symptoms, physical activity (PA), and adipose tissue hormones [27–31]. Further details about these reports can be found in Table 3.

Both the crossover RCT and case report observed weight gain in participants with AN treated with dronabinol [27, 31]. When participants from the crossover RCT received 5 mg treatment, they gained an average of 1 kg over 4 weeks, but only gained an average of 0.34 kg over 4 weeks when they received a placebo (p = 0.03 for difference) [27]. In the case report, the participant’s BMI increased from 19.5 to 21.0 kg/m^2^ over 6 weeks of 15 mg treatment [31]. On the other hand, the non-randomized study did not find significant weight changes after 2 mg treatment lasting 4 weeks [30].

The non-randomized study and case report observed improved ED symptoms in participants with AN during cannabinoid treatment [30, 31]. Participants from the non-randomized study reported significantly reduced ascetism and increased body care using the Eating Disorder Inventory and Eating Attitude Test, and significantly reduced depression using Beck Depression Inventory during 4 weeks of 2 mg treatment [30]. The participant from the case report reported reduced ED symptoms from items included in the Eating Disorder Examination Questionnaire over 6 weeks of 15 mg treatment [31]. Contrary to these findings, the crossover RCT found no significant changes in ED symptoms using the Eating Disorder Inventory-2 and the non-randomized study reported no significant changes in anxiety using the Spielberger State-Trait Anxiety Inventory, one of which involved 5 mg treatment lasting 4 weeks [27], while the other involved 2 mg treatment lasting 4 weeks [27, 30].

PA levels are often elevated in those with AN and contribute to maintenance of low weight status [33]. The crossover RCT found significant differences in PA during 5 mg treatment lasting 4 weeks based upon whether participants were inpatients or outpatients [28]. Dronabinol resulted in significant increases in the duration of PA in outpatients and significant increases in the intensity of PA in inpatients [28]. Conversely, in the case report, the participant’s PA urge and number of steps taken per day decreased during 15 mg treatment lasting 6 weeks [31].

Altered adipose tissue hormones, including, low leptin levels, low cortisol levels, high adiponectin levels, and low insulin-like growth factor (IGF) proteins are common in individuals with AN [34]. Both the crossover RCT (5 mg treatment lasting 4 weeks) and case report (15 mg treatment lasting 6 weeks) found no changes in leptin and an increase in cortisol during dronabinol treatment [29, 31]. Only the crossover RCT (5 mg treatment lasting 4 weeks) measured adiponectin and IGF proteins and found significantly lowered adiponectin when controlling for leptin levels and no significant changes in IGF proteins [29].

Benefits of cannabinoid treatment included weight gain, reduced ED symptoms, and reduced PA [27, 30, 31]. However, null effects/harms were also observed, including no changes in weight, ED symptoms, or adipose tissue hormones, and increased PA [27–31].

### Harms of cannabis use

Four reports from 4 studies (2 retrospective cohort studies and 2 case reports) reported harms associated with cannabis use in AN [35–38]. Both participants from the case reports identified as women while the gender/sex breakdown of participants with AN could not be determined from the data included in the retrospective cohort studies. Sample sizes ranged from 1 to 16,922 participants, although the exact sample size of participants with AN from one of study could not be determined [35]. Outcomes of interest included genetic factors related to cannabis use disorder (CUD) in AN and associations between compensatory behaviors and cannabis abuse/dependence. Further details about these reports can be found in Table 3.

Two retrospective cohort studies assessed polygenic risk scores (PRS) for AN and CUD, 1 of which found that the highest PRS for AN were significantly associated with a higher risk of CUD [35], while the other did not find significant associations between PRS for AN and PRS for CUD [36].

Two case reports, each involving 1 participant with binge/purge type AN reported compensatory behaviors following cannabis use [37, 38]. One of these participants was using cannabis daily for at least 7 years [37], while the other participant was using cannabis daily for at least 3 years [38]. One participant presented to a treatment facility and was found to be suffering from cannabinoid hyperemesis syndrome (CHS), a condition associated with long term cannabis use causing recurrent vomiting [13, 37]. Although the other participant did not have CHS, appetite stimulation from cannabis appeared to encourage the participant to engage in postprandial compensatory behaviors to maintain low weight status, which was supported by a reduced BMI over the span of 3 years of daily use [38].

## Discussion

This scoping review presents results from literature examining differences in the ECS between those with AN and HC, the benefits/harms/null effects of cannabinoid treatment on AN symptoms, and harms of cannabis use for those with AN. A range of study designs were represented: crossover RCT, non-randomized trial, retrospective cohort, case control, combined cross-sectional longitudinal, pre-meal/post-meal, cross-sectional, and case report.

### Endocannabinoid system

Studies that explored the ECS showed differences in genetic factors such as alleles that code for receptors within the ECS in AN, higher availability of the CB1 receptor in AN, and differences in levels of circulating endocannabinoids in AN compared to HC [17–24]. Furthermore, some studies found associations between abnormally elevated/lowered receptors and endocannabinoids and AN symptoms, such as lower weight and higher emotional dysregulation [20, 24]. Together, these findings propose the possibility that the ECS may be involved with the pathophysiology of AN (based on n = 14–763 participants with AN). Studies included in this review align with an existing review which propose that components of the ECS may serve as a possible biomarker for AN [10]. However, the current state of the research does not point to one particular biomarker in the ECS due to inconsistencies between studies and their results. For example, 1 study that explored circulating endocannabinoids found elevated levels of AEA [21] while others found lowered levels of AEA in AN compared to HC [22, 23]. This and other inconsistencies are likely attributable to differences in methodology, as some studies controlled for factors such as time since last meal or hedonic eating, both of which impact circulating endocannabinoid levels [26], while others did not. Therefore, controlling for these and other factors are important considerations for future research. Longitudinal designs that follow participants with AN throughout weight restoration are needed to understand changes in endocannabinoid levels, as data from 1 study proposed the possibility that endocannabinoids begin to approach levels similar to that of HC once weight is restored (based on n = 67 participants falling under the UWAN category) [21]. Given this proposed relationship between weight changes and endocannabinoids, future studies might consider measuring endocannabinoids in individuals with AN and atypical AN. Although individuals with atypical AN fall within BMI categories of normal or above, they have experienced significant weight loss, the same psychological symptoms, and may also experience physiological manifestations of AN [39]. The question of whether endocannabinoids in atypical AN differ from AN and HC, and if they change during recovery is a fertile avenue for future research.

### Benefits/harms/null effects of cannabinoid treatment

Cannabinoid treatment was associated with beneficial effects for AN, including weight gain, improved ED symptoms, and reduced PA urge; however, harms/null effects such as increased PA, were also associated with treatment [27–31]. The dose range of cannabinoid treatment (2–15 mg), could be a contributor to inconsistencies between studies and their results. In some instances, both lower dosages and higher dosages showed similar results. Both a study that administered 2 mg [30] and a study that administered 15 mg [31] found reduced ED symptoms. In other instances, dosages within similar ranges showed different results. A study that administered 2 mg [30] found no changes in weight while a study that administered 5 mg [27] found significant weight gain. Sample sizes used in treatment studies ranged from 1–24 participants. Inconsistent findings could be attributable to insufficient power to detect an effect in the case of null findings, or a wide variability of individual differences in response to cannabinoids. It is possible that individualized dosing is needed to maximize beneficial effects and minimize harmful effects.

Individualized dosing should consider cannabinoids’ effects. THC is known to promote appetite which can be helpful for weight gain in individuals with AN [7]. However, in excess, THC can induce anxiety, which would be counterproductive for AN recovery [40]. This may lead to the assumption that low doses of THC/dronabinol would show the best results for AN (i.e., appetite stimulation without anxiety). Indeed, the lowest dose of THC/dronabinol used for AN was 2 mg, which was associated with reduced ED symptoms [30]. However, the highest dose of THC/dronabinol used for AN was 15 mg, which was also associated with reduced ED symptoms [31]. Given these data, the question of whether THC affects individuals differently based on characteristics that have not yet been explored is a fertile avenue for future research. Of note, none of the studies included in this scoping review administered the other major cannabinoid, CBD. CBD is known to reduce anxiety, which is a symptom of AN [40, 41]. In combination with CBD, THC may induce appetite without worsening anxiety, which would be ideal for AN. Future studies may wish to consider the benefits and risks of both THC and CBD, to optimize positive outcomes for AN.

### Harms of cannabis use

Literature regarding harms associated with cannabis use puts forth the possibility that individuals who are genetically predisposed to developing AN may also be genetically predisposed to developing CUD [35]. Additionally, cannabis use may contribute to engagement in compensatory behaviors in some individuals with binge/purge type AN [35, 37, 38]. As established by an existing review, cannabis abuse/dependence is more common in binge/purge type AN [12]. The present review expands upon this finding as it reports additional harms of cannabis use from 2 individuals with binge/purge type AN [37, 38]. While limited by sample size and study design, these case reports proposed the possibility that cannabis use may coincide with compensatory behaviors. While 1 study suggested that the appetite-inducing effects of cannabis encouraged compensatory behaviors [38], the other suggested that CHS could be overlooked in those with binge/purge type AN due to similarities in symptoms [37]. If these results are replicated in future studies, it is important to track whether cannabis use may lead to weight loss, or other counterproductive situations for AN recovery. Future studies exploring cannabinoid treatment may wish to consider including measures for compensatory behaviors and problems associated with use.

### Future research/clinical implications

Further investigation with consistent methodology may provide a clearer understanding of how endocannabinoids present in AN and the associations they may have with psychological and physiological symptoms of AN. Furthermore, understanding how endocannabinoids are affected throughout recovery may provide guidance for how to best target the ECS using exogeneous cannabinoids. Dose–response studies exploring THC/dronabinol should be conducted to determine the most appropriate dose for individuals with AN, or if an individualized approach is needed to maximize beneficial effects and minimize harmful effects. Furthermore, RCTs may wish to add CBD to their cannabinoid regimens to better approximate the effects of the major cannabinoids found in the marijuana plant, as each plays a unique role.

### Strengths and limitations

Because research on cannabis and AN is rather new, it is important to consider all levels of evidence, which the present scoping review did. At the same time, factors influencing bias, such as study design were not evaluated, as this level of assessment is not consistent with the aims of a scoping review [15]. Since this review excluded reports published in languages other than English, it is possible that pertinent evidence was excluded. It is also important to note that conclusions drawn in this review were based upon studies that included sample sizes ranging from 1- 16,992 participants, which limits generalizability in cases of small sample sizes. Lastly, none of the reports included participants with atypical AN, making it difficult to understand whether results included in this review also apply to those with atypical AN.

## Conclusion

This scoping review provides an overview of studies exploring the ECS, benefits/harms/null effects of cannabinoid treatment, and harms of cannabis use for those with AN. Although results between studies exploring the ECS were inconsistent, circulating endocannabinoids appear to be different in those with AN compared to HC and may be associated with psychological and physiological AN symptoms. While these results propose biological justification for cannabinoid treatment for AN, and some results from treatment studies showed beneficial outcomes such as weight gain and reduced ED symptoms, other results suggested ineffectiveness of cannabinoid treatment for AN, and some propose potential harms. This body of research is limited in scope, presenting avenues for further investigation including relationships between circulating endocannabinoids and AN recovery, and individualized dosing that may include both THC and CBD, to maximize benefits and minimize potential harms.

### Supplementary Information

**Additional file 1.** PRISMA-ScR Checklist.

**Additional file 2.** Search Terms.

## Data Availability

Not applicable.

## Abbreviations

AN: Anorexia Nervosa
ED: Eating Disorder
THC: ∆9-Tetrahydrocannabinol
CBD: Cannabidiol
ECS: Endocannabinoid System
CB1/CB2: Cannabinoid Receptors 1 and 2
AEA: N-arachidonoylethanolamine
2-AG: 2-Arachidonoylglycerol
CHS: Cannabinoid Hyperemesis Syndrome
RCT: Randomized Controlled Trial
HC: Healthy Controls
PRISMA-ScR: Preferred Reporting Items for Systematic Reviews and Meta-Analyses extension for Scoping Reviews
MeSH: Medical Subject Headings
PCC: Population Concept Context
BMI: Body Mass Index
OEA: Oleoylethanolamide
PEA: Palmitoylethanolamide
SEA: Stearoylethanolamide
UWAN: Underweight AN
WRAN: Weight restored AN
CI: Confidence Interval
NS: Not Significant
RAN: Restrictive type AN
BPAN: Binge/Purge type AN
T1: Cross-sectional period
T2: Longitudinal period
PA: Physical Activity
IGF: Insulin-like growth factor
CUD: Cannabis use Disorder
PRS: Polygenic Risk Scores
IGFBP: Insulin-like growth factor binding protein
