# Supplementary material for: The relationship between cannabis and anorexia nervosa: a scoping review
Source: J Eat Disord. 2023 Oct 19;11:186. doi: 10.1186/s40337-023-00887-9 (PMC10585887; doi:10.1186/s40337-023-00887-9)
Supplement: Supplementary file 2 — Additional file 2. Search Terms. [file 40337_2023_887_MOESM2_ESM.docx]

PubMed:

"Cannabis"[Mesh] OR "Dronabinol"[Mesh] OR Cannabis OR Dronabinol OR Cannabinol OR Marinol OR Δ9-Tetrahydrocannabinol OR Cannabidiol OR THC OR CBD OR Cannabinoids OR Marijuana OR "Endocannabinoids"[Mesh] OR "Receptors, Cannabinoid"[Mesh] OR Endocannabinoids OR Endocannabinoid system OR ECS OR Cannabinoid receptors OR Cannabinoid receptor 1 OR cannabinoid receptor 2 OR CB1 OR CB2 OR arachidonoylglycerol OR anandamide OR oleoylethanolamide OR palmitoylethanolamide OR "Marijuana Abuse"[Mesh] OR Cannabis abuse OR cannabis use disorder OR CUD OR cannabis dependence OR marijuana abuse OR marijuana dependence OR (cannabis AND dangers) OR (cannabis AND harms) OR (cannabis AND adverse effects) OR (cannabis AND negative effects) AND ("Anorexia Nervosa"[Mesh] OR Primary anorexia OR Anorexia Nervosa OR Anorexia OR Atypical anorexia nervosa OR Atypical anorexia)

PsycINFO:

(Cannabis OR Dronabinol OR Cannabinol OR Marinol OR Δ9-Tetrahydrocannabinol OR Cannabidiol OR THC OR CBD OR Cannabinoids OR Marijuana OR Endocannabinoids OR Endocannabinoid system OR ECS OR Cannabinoid receptors OR Cannabinoid receptor 1 OR cannabinoid receptor 2 OR CB1 OR CB2 OR arachidonoylglycerol OR anandamide OR oleoylethanolamide OR palmitoylethanolamide OR Cannabis abuse OR cannabis use disorder OR CUD OR cannabis dependence OR marijuana abuse OR marijuana dependence OR cannabis harms OR cannabis adverse effects OR cannabis negative effects) AND (Primary anorexia OR Anorexia Nervosa OR Anorexia OR Atypical anorexia nervosa OR Atypical anorexia)

Web of Science:

TS=(Cannabis OR Dronabinol OR Cannabinol OR Marinol OR Δ9-Tetrahydrocannabinol OR Cannabidiol OR THC OR CBD OR Cannabinoids OR Marijuana OR Endocannabinoids OR “Endocannabinoid system” OR ECS OR “Cannabinoid receptors” OR “Cannabinoid receptor 1” OR “cannabinoid receptor 2” OR CB1 OR CB2 OR arachidonoylglycerol OR anandamide OR oleoylethanolamide OR palmitoylethanolamide OR “Cannabis abuse” OR “cannabis use disorder” OR CUD OR “cannabis dependence” OR “marijuana abuse” OR “marijuana dependence” OR "cannabis harms" OR "cannabis adverse effects" OR "cannabis negative effects") AND TS=(“Primary anorexia” OR “Anorexia Nervosa” OR Anorexia OR “Atypical anorexia nervosa” OR “Atypical anorexia")

Cochrane:

#1 MeSH descriptor: [Cannabis] explode all trees 507

#2 MeSH descriptor: [Dronabinol] explode all trees 902

#3 MeSH descriptor: [Endocannabinoids] explode all trees 87

#4 MeSH descriptor: [Receptors, Cannabinoid] explode all trees 106

#5 MeSH descriptor: [Marijuana Abuse] explode all trees 774

#6 #1 OR #2 OR #3 OR #4 OR #5 1972

#7 (Cannabis OR Dronabinol OR Cannabinol OR Marinol OR Delta 9 Tetraydrocannabinol OR Cannabidiol OR THC OR CBD OR Cannabinoids OR Marijuana OR Endocannabinoids OR Endocannabinoid system OR ECS OR Cannabinoid receptors OR Cannabinoid receptor 1 OR cannabinoid receptor 2 OR CB1 OR CB2 OR arachidonoylglycerol OR anandamide OR oleoylethanolamide OR palmitoylethanolamide OR Cannabis abuse OR cannabis use disorder OR CUD OR cannabis dependence OR marijuana abuse OR marijuana dependence OR (cannabis AND danger) OR (cannabis AND harms) OR (cannabis AND adverse effects) OR (cannabis AND negative effects)):ti,ab,kw 7022

#8 #6 OR #7 7022

#9 MeSH descriptor: [Anorexia Nervosa] explode all trees 727

#10 (Primary anorexia OR Anorexia Nervosa OR Anorexia OR Atypical anorexia nervosa OR Atypical anorexia):ti,ab,kw 6198

#11 #9 OR #10 6198

#12 #8 AND #11 77
